# Role and Therapeutic Targeting of SDF-1α/CXCR4 Axis in Multiple Myeloma

**DOI:** 10.3390/cancers13081793

**Published:** 2021-04-09

**Authors:** Shigeki Ito, Tsuyoshi Sato, Takahiro Maeta

**Affiliations:** Division of Hematology & Oncology, Department of Internal Medicine, Iwate Medical University School of Medicine, Yahaba 028-3695, Japan; satotsu@iwate-med.ac.jp (T.S.); maetatak@iwate-med.ac.jp (T.M.)

**Keywords:** CXCR4, SDF-1α, multiple myeloma, drug resistance, extramedullary disease

## Abstract

**Simple Summary:**

The SDF-1α/CXCR4 axis plays crucial roles in proliferation, survival, invasion, dissemination, and drug resistance in multiple myeloma. This review summarizes the pleiotropic role of the SDF-1α/CXCR4 axis in multiple myeloma and introduces the SDF-1α/CXCR4 axis-targeted therapies in multiple myeloma.

**Abstract:**

The C-X-C chemokine receptor type 4 (CXCR4) is a pleiotropic chemokine receptor that is expressed in not only normal hematopoietic cells but also multiple myeloma cells. Its ligand, stromal cell-derived factor 1α (SDF-1α) is produced in the bone marrow microenvironment. The SDF-1α/CXCR4 axis plays a pivotal role in the major physiological processes associated with tumor proliferation, survival, invasion, dissemination, and drug resistance in myeloma cells. This review summarizes the pleiotropic role of the SDF-1α/CXCR4 axis in multiple myeloma and discusses the future perspective in the SDF-1α/CXCR4 axis-targeted therapies in multiple myeloma.

## 1. Introduction

Multiple myeloma (MM) is the second most common hematological malignancy. The availability of new drugs (e.g., proteasome inhibitors, immunomodulatory drugs, monoclonal antibodies, and histone deacetylase inhibitors (HDACi)) has greatly advanced the treatment and improved the survival of patients with MM over the last two decades [1,2]. However, the disease eventually relapses in most of the patients. Thus, identifying novel molecular targets and developing new therapeutic agents are urgently required to further improve the prognosis of patients with MM.

Stromal cell-derived factor-1α (SDF-1α) is a homeostatic chemokine produced in bone marrow stromal cells (BMSCs) [3]. SDF-1α was initially discovered as a pre-B cell growth factor that is indispensable for homeostatic processes (e.g., lymphopoiesis and embryogenesis) [4]. Additionally, SDF-1α is an essential factor in physiological and pathological processes, including embryogenesis, hematopoiesis, angiogenesis, and inflammation [5]. Consequently, SDF-1α is responsible for hematopoietic stem cells (HSCs) and progenitor cells (HPCs) retention in the bone marrow (BM) [6,7]. It stimulates the migration and homing of HSCs and HPCs via the G protein-coupled receptor C-X-C chemokine receptor type 4 (CXCR4) [8]. SDF-1α or CXCR4 knockout mice are with embryonic lethality because each gene knockout impaired hematopoiesis due to a defect in the trafficking of HSCs from the fetal liver to the embryonic BM, defects in the heart and brain development, and vascularization [4,9,10]. The SDF-1α/CXCR4 axis is also involved in physiological processes including angiogenesis, metastasis, and survival in malignant tumors [11,12,13].

The SDF-1α/CXCR4 axis plays a pleiotropic role in the expansion and colonization of MM cells in the BM [14] and in the homing, adhesion, invasion, migration, and mobilization of MM cells out of the BM [14]. CXCR4 expression is present in approximately 60% of primary MM cells from the BM and is associated with disease activity [15]. Moreover, elevated SDF-1α serum levels are correlated with an increased osteolytic disease [16]. A deeper understanding of the roles of the SDF-1α/CXCR4 axis in MM is necessary for the identification of novel molecular targets and the development of newer drugs and treatment strategies. In this review, the pleiotropic role of SDF-1α/CXCR4 axis and SDF-1α/CXCR4 axis-targeted therapies in MM is summarized.

## 2. The Role of SDF-1α/CXCR4 in Hematopoiesis

SDF-1α and its CXCR4 receptor are involved in the hematopoiesis regulation at the HSC and HPC levels. SDF-1α is a crucial ligand for homing, retention, and survival of HSCs and HPCs in the BM microenvironment [6,17,18,19]. Hence, inhibiting the SDF-1α and CXCR4 interaction leads to enhanced mobilization of HSCs and HPCs to the blood. A specific antagonist of SDF-1α binding to CXCR4, AMD3100 (plerixafor), has been clinically used for inducing HSCs and HPCs mobilization in humans [20,21,22,23,24]. It synergizes with granulocyte colony-stimulating factor (G-CSF) to greatly enhance G-CSF-induced mobilization of HSCs and HPCs [25,26,27]. Moreover, adhesion molecules have been also implicated in homing of HSCs and HPCs [28]. The integrin very late antigen (VLA)-4 mediates HPCs adhesion to BMSCs by interacting with connecting segment-1 (CS-1)/fibronectin and vascular cell adhesion molecule-1 (VCAM-1). SDF-1α rapidly and transiently upregulated the adhesion of CD34^+^ BM cells to both CS-1/fibronectin and VCAM-1, and BM stromal cells, suggesting that SDF-1α could modulate VLA-4-mediated CD34^+^ BM cell adhesion [28].

## 3. Role of SDF-1α/CXCR4 in MM

**Figure 1:**
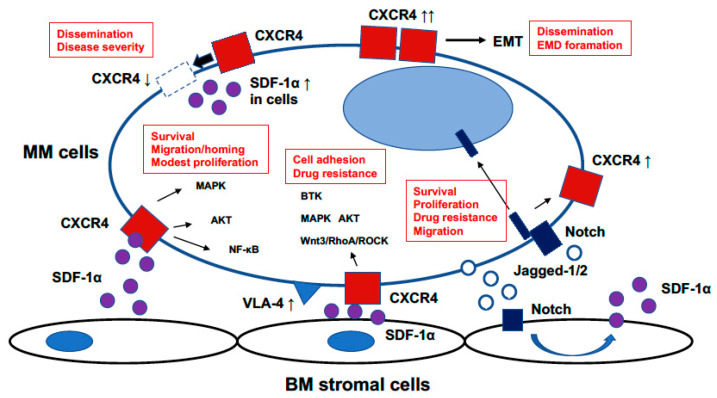
Pleiotropic roles of Stromal cell-derived factor-1α (SDF-1α)/C-X-C chemokine receptor type 4 (CXCR4) axis in multiple myeloma cells. SDF-1α/CXCR4 axis plays pleiotropic roles in multiple myeloma (MM) pathogenesis including proliferation, survival, migration, homing, drug resistance, epithelial-mesenchymal transition (EMT), dissemination, and extramedullary disease (EMD) formation.

The SDF-1α/CXCR4 axis plays a critical role in proliferation, survival, invasion, dissemination, metastasis, and drug resistance in MM cells [29]. Additionally, SDF-1α levels in BM plasma and BM stromal cell culture supernatant were elevated in patients with MM [30]. Binding SDF-1α to CXCR4 activates a variety of intracellular signaling pathways that regulate these biological processes. Furthermore, SDF-1α induces MEK1/2, p42/44 MAPK, and AKT phosphorylation in MM cell lines and patient MM cells [30] and activates nuclear factor-κB [31,32]. Hideshima et al. showed that SDF-1α induced modest increases in proliferation in both MM cell lines and primary MM cells [30]. A previous study also showed that SDF-1α did not affect proliferation and survival in lymphohematopoietic cells [33]. SDF-1α is produced by not only BMSCs or vascular endothelial cells but also circulating plasma cells (cPCs) in MM. Recently, Geng et al. demonstrated that SDF-1α was abnormally upregulated in cPCs using single-cell transcriptome analysis [34]. Furthermore, Martin et al. reported that high levels of SDF-1α produced by MM plasma cells promoted osteolysis and bone marrow angiogenesis [16,35]. These findings suggest that tumor cell-generating SDF-1 plays critical roles in osteolysis and angiogenesis in MM, and that abnormal SDF-1 auto-secretion may contribute to cPC extramedullary translocation from the BM. This section reviews the role of CXCR4 on mobilization, drug resistance, Notch pathway, and dissemination in MM (Figure 1).

### 3.1. Roles of SDF-1α/CXCR4 on Mobilization in MM

High-dose chemotherapy followed by autologous stem cell transplantation (ASCT) is a standard therapy in transplant-eligible patients with MM [36]. Furthermore, HSC mobilization and collection have been performed via chemotherapy administration, hematopoietic growth factors administration (including G-CSF or GM-CSF), or chemotherapy plus hematopoietic growth factor [37,38,39,40]. Plerixafor, a small-molecule bicyclam, reversibly binds to CXCR4 and antagonizes SDF-1α interaction. It was approved by the Food and Drug Administration in 2008 and widely used in several countries as a mobilizer for harvesting HSCs and HPCs [21,22,23,24,25]. Plerixafor is effective in the mobilization of peripheral blood stem cells when combined with G-CSF [41]. The AMD3100-3102 study was a multicenter randomized, double-blind, placebo-controlled trial, evaluating the safety and efficacy of plerixafor plus G-CSF versus placebo plus G-CSF in mobilizing HSCs in MM patients. The results showed that plerixafor and G-CSF were well-tolerated and significantly more patients could harvest the optimal CD34^+^ cells/kg compared with G-CSF alone [41]. Plerixafor plus G-CSF has been widely used for harvesting HSCs in transplant-eligible patients with MM based on these results. Consequently, the comobilization of MM cells following chemotherapy or growth factors has been documented [37,42,43,44,45]. Therefore, the SDF-1α/CXCR4 axis and adhesion molecules play crucial roles in homing and mobilization of not only HSCs but also MM cells [46]. A significant decrease in SDF-1α plasma levels and CXCR4 expression on MM cells in the apheresis product compared with those in BM before mobilization was observed. Additionally, decreased VLA-4 expression was observed on MM cells in the apheresis product. These findings suggest that mobilization of MM cells involves SDF-1α/CXCR4 signaling and downregulation of VLA-4 expression [46]. Alsayed et al. demonstrated that CXCR4 is expressed at high levels on the surface of MM cells in the peripheral blood compared with those in the BM. Furthermore, SDF-1α levels were markedly elevated in the BM of MM patients compared with those in the peripheral blood of MM patients and those in the peripheral blood and the BM samples of healthy controls, suggesting that CXCR4 is downregulated in the BM in response to high SDF-1α levels. Additionally, AMD3100 inhibited the migration and homing of MM cells in vitro and in vivo [14]. Moreover, Hideshima et al. showed that SDF-1α induces migration of MM cells although the effect is modest [30]. Hence, CXCR4 functions in the mobilization of MM cells. Plerixafor may mobilize MM cells and contaminate MM cells in apheresis product, contributing to disease relapse after ASCT in the clinical setting. Nahi et al. reported a randomized, phase II study evaluating MM cell mobilization and apheresis product contamination in patients treated with G-CSF alone or plerixafor plus G-CSF [47]. The primary endpoint was the number of MM cells in the peripheral blood and apheresis product after the administration of G-CSF + plerixafor versus G-CSF alone. Patients in whom a partial response (PR) or better was obtained after induction therapy and those in whom the percentage of MM cells in BM was <10% before mobilization was included in this study. The threshold of myeloma cell contamination in the apheresis product was defined as 4.5 × 10^5^ MM cells/kg body weight because the value is considered to be sufficient to restart the tumor growth when apheresing the CD34^+^ cells [48]. No patient with MM cells in the peripheral blood up to day eight of G-CSF administration in either treatment group was noted. Additionally, it was noted that no patients in the G-CSF + plerixafor group and only one patient in the G-CSF group mobilized at least 4.5 × 10^5^ MM cells in the apheresis product up to day eight. G-CSF + plerixafor administration does not affect the number of MM cells mobilized in patients who achieved at least PR and in whom BM involvement was <10%. The plerixafor could be used safely because most MM patients obtain a deeper response in the induction therapy era with novel agents. Therefore, further study to elucidate the impact on survival in patients who received plerixafor as a mobilizer is warranted.

### 3.2. Roles of SDF-1α/CXCR4 on Drug Resistance in MM

Almost all MM patients eventually relapse and become refractory to multiple drugs despite recent advances in drug development and the introduction of novel agents [49,50]. The drug resistance mechanism is not fully elucidated although extensively studied. Cellular adhesion-mediated drug resistance (CAM-DR) is one of the underlying mechanisms of disease relapse and refractoriness to antimyeloma therapy [51]. Elucidating the mechanism of CAM-DR and developing the corresponding drugs are urgently required to improve outcomes. MM cells express several mediators of cellular adhesion, including CD44, VLA-4, and CXCR4 [52]. The SDF-1α/CXCR4 pathway plays an essential role in cellular adhesion [46,53]. BMSCs produce several adhesion molecules, cytokines, and chemokines such as SDF-1α which are necessary for the proliferation and survival of MM cells. These promote the adhesion between MM cells and BMSCs, thereby inducing drug resistance [54,55]. The coculture of MM cells with BMSCs increased the drug resistance and suppressed the cell death of MM cells. Consequently, Liu et al. demonstrated that SDF-1α-induced interleukin-6 (IL-6) upregulation-mediated drug resistance and apoptosis of MM cell lines in the adhesion state [56]. The report mentioned that SDF-1α treatment-induced PI3K and AKT phosphorylation in MM cells. Furthermore, several other signaling pathways including MAP/ERK [14,57], Wnt3/RhoA/ROCK [58], and HMG-CoA/Rho/Rho-kinase [59] are involved in CAM-DR of MM.

Bruton’s tyrosine kinase (BTK) is a regulator of myeloma stemness and senescence and is related to MM progression and drug resistance [60,61]. Consequently, BTK expression was correlated with CXCR4 surface expression [62]. Additionally, ibrutinib, a BTK inhibitor, could reduce the surface membrane levels of CXCR4 in chronic lymphocytic leukemia and downregulate the migration of MM cells toward SDF-1 and homing to the BM microenvironment [63,64]. Furthermore, Wang et al. recently demonstrated that BTK induces CAM-DR through CXCR4 regulation degradation in MM [64], promoting BTK expression induced MM cell adherence to the extracellular matrix and stromal cells in vitro and in vivo and increased drug resistance to bortezomib and doxorubicin in MM cells. Treatment with BTK inhibitor showed synergistic effects with bortezomib in mouse models. Hence, BTK bound directly with CXCR4 and prevented its ubiquitination-induced degradation, leading to CAM-DR maintenance. BTK plays a role in CAM-DR through the regulation of CXCR4 degradation in MM cells and suggests that targeting therapy for the BTK/CXCR4 interaction may be effective for reversing CAM-DR.

Pan-HDACi panobinostat is clinically used in combination with bortezomib or lenalidomide and dexamethasone for relapsed and refractory MM [65,66]. However, panobinostat lacks therapeutic activity as a single agent. Beider et al. found that sensitivity of MM cells and primary MM cells to panobinostat was associated with decreased CXCR4 expression, whereas CXCR4 overexpression increased their resistance to panobinostat [67]. Additionally, CXCR4 overexpression led to mammalian target of rapamycin (mTOR) activation in response to panobinostat treatment in MM cells, suggesting that mTOR pathway activation induces resistance to panobinostat. Combining panobinostat with mTOR inhibitor everolimus was also shown to abrogate HDACi resistance and induced synergistic cell death. These results provide the rationale for a novel treatment strategy to overcome CXCR-4-mediated resistance to HDACi in MM. Waldschmidt et al. also evaluated CAM-DR restoration using different antimyeloma agents and the CXCR4 inhibitor plerixafor [52]. Moreover, the plerixafor reduced the VLA-4 and CD44 expressions, both of which are known as essential mediators of BM adhesion on MM cells. Consequently, the plerixafor restored sensitivity to bortezomib and pomalidomide in stromal cell coculture.

The SDF-1α/CXCR4 pathway plays a crucial role in drug resistance in MM. Developing a therapeutic strategy against this pathway is required to improve MM outcomes.

### 3.3. Notch and SDF-1α/CXCR4 in MM

Notch plays a role in myeloma pathophysiology. Moreover, Notch receptors are transmembrane proteins that are activated by specific ligands including Jagged-1,-2 and Delta-Like-1,-3,-4 [68]. The binding of ligands to the receptors introduces the γ-secretase-mediated release of the intracellular domain which in turn translocates to the nucleus and activates target genes [69]. MM cells express Notch-1, Notch-2, and Notch-3 and the ligands Jagged-1 and Jagged-2 [70,71,72,73,74,75]. Additionally, Notch signaling is activated by their homotypic interaction in MM cells. Notch activation in MM cells leads to inhibition of apoptosis, drug resistance, and increased osteolysis [76,77,78]. Moreover, Notch-1 and Jagged-1 expression are related to disease progression from the monoclonal gammopathy of undetermined significance to the MM [70]. Additionally, Jagged-2 is overexpressed in MM patients [71,74,75]. However, the relationship between the Notch pathway and SDF-1α/CXCR4 axis has not been fully elucidated. Mirandola et al. investigated the association between Notch receptors and the SDF-1α/CXCR4 axis [79]. Notch was shown to positively control not only CXCR4 but also SDF-1α expression and to function in MM cell lines. Moreover, the inhibition of Notch signaling was found to prevent MM cell migration, proliferation, and resistance to apoptosis through reducing CXCR4 and SDF-1α levels. Colombo et al. recently demonstrated that myeloma cell-derived Jagged-1 and Jagged-2 triggered Notch activity in BMSCs [80]. These Jagged ligands secrete higher levels of SDF-1α in the BM microenvironment, increasing CXCR4 activation in myeloma cells. Additionally, SDF-1α induced Bcl-2, survivin, and ATP binding cassette subfamily C member 1 (ABCC1) expression. Moreover, the Jagged inhibition was shown to cause a decrease in both myeloma-intrinsic and stromal cell-induced resistance to antimyeloma drugs including bortezomib, lenalidomide, and melphalan. These findings indicate that Notch may have a role not only in MM progression but also in drug resistance through regulating the SDF-1α/CXCR4 axis and providing the proof of concept that targeting strategy for Jagged/Notch pathway in MM cells and BM stromal cells could restore drug resistance.

### 3.4. SDF-1α/CXCR4 in Extramedullary Disease

The proliferation of malignant plasma cells is restricted in the BM in most patients with MM. However, extramedullary disease (EMD) occurs in a subset of patients. Generally, the disease involves the soft tissue, cortical bone, and central nervous system. Approximately 7–18% of the patients have EMD at the time of initial diagnosis [81,82,83,84] and 20% of the patients develop EMD later in the course of the disease [81,82,84]. The EMD development was reported to be associated with poor prognosis in patients with MM [83,84,85]. However, these studies were performed before the introduction of novel agents. Lee et al. evaluated the prognostic impact of EMD on newly diagnosed MM in the context of treatment approaches in the era of novel agents including ASCT and chemotherapy alone. The presence of EMD at diagnosis was demonstrated to be associated with worse progression-free survival (PFS) and overall survival (OS) compared with those without EMD at diagnosis [86]. The presence of EMD at diagnosis was an independent prognostic factor for PFS and OS in transplant-ineligible patients but not in transplant-eligible patients. Additionally, the adverse impact of EMD observed in transplant-ineligible patients was attenuated among the patients with bortezomib. These findings indicate that ASCT can overcome the negative impact of EMD, and bortezomib has activity on EMD in transplant-ineligible patients [86]. Elucidating the mechanism underlying extramedullary spread and developing newer treatment approaches is essential to improve outcomes of MM with EMD.

The possible mechanism of extramedullary myeloma spread has not been fully understood although proposed. In one possible EMD development mechanism, metastatic MM cells initially exit the BM, translocate into the blood as clonal circulating plasma cells (cPCs), and finally settle in the peripheral tissues and develop an EMD [87,88,89]. Vande Broek et al. showed that the downregulation of chemokine receptors including CXCR4 has been observed in patients with active diseases compared with those with nonactive diseases [15]. Additionally, Olivera et al. showed that thalidomide exposure induces the downregulation of SDF-1α and CXCR4 in MM patients [90]. Consequently, thalidomide treatment could facilitate extramedullary spread and growth. Geng et al. explored the transcriptomic differences between MM cells in BM and peripheral cPCs in each patient with EMD using single-cell RNA sequencing. SDF-1α and CXCL7, which is also known as another BM attracting chemokine, are abnormally upregulated in cPCs and were also found [34]. The findings suggest that both chemokines produced by MM cells may contribute to the initial extramedullary translocation of cPCs from BM and the eventual formation of EMD. Stessman et al. showed that bortezomib-resistant (BzR) cells displayed a decreased affinity for the BM compartment compared with bortezomib-sensitive (BzS) cells and more extramedullary spread in mouse models [91]. A loss in CXCR4 mRNA expression in BzR cells was found compared with BzS cells. Low CXCR4 expression was associated with poor outcomes in patients treated with bortezomib in both the APEX trial [92] and MM total therapy 3 (TT3) trial [93]. These findings suggest that decreased CXCR4 expression is associated with increased disease severity.

Epithelial–mesenchymal transition (EMT) plays a crucial role in both physiological conditions and pathological settings [94,95], as well as cancer progression and metastasis [96,97]. CXCR4 and SDF-1α have been reported to act as positive regulators of tumor cell metastasis in solid tumors [98,99]. Roccaro et al. demonstrated that CXCR4 enhanced the acquisition of an EMT-like phenotype in MM cells and induced higher bone metastasis and EMD dissemination in vivo [100]. EMD- and BM-prone MM cells were generated by in vivo selection approach. EMD-prone clone colonized BM niche and could be metastasized to and engraft within extramedullary sites, whereas extramedullary infiltration of the BM clone was not detectable. The transcriptional profile by RNA sequencing included genes defining an EMT, hypoxia-associated genes, and TNFα/NF-κB response-related genes. These findings indicated that EMT occurs not only in BM-disseminating MM cells but also in MM cells colonizing EMD sites. Additionally, both BM- and EMD-prone clones expressed higher surface CXCR4 compared with the parental cells. Moreover, the CXCR4 expression in the EMD-prone cells was relatively higher compared with the BM-prone cells. CXCR4 enhanced the acquisition of an EMT-like phenotype in MM cells with a phenotype conversion for invasion. Ulocuplumab, a monoclonal anti-CXCR4 antibody, suppressed MM cell dissemination, suggesting the inhibition of EMT-like phenotype conversion of MM cells by targeting CXCR4 [100]. These findings strongly suggest that SDF-1α and CXCR4 play a central role in MM disease progression and EMD development.

## 4. SDF-1α/CXCR4 Targeted Therapy in MM

Molecular-targeted antimyeloma drug candidates have been developed on the basis of the molecular pathological findings of the SDF-1α/CXCR4 axis in MM. This section introduces developing drugs targeting the SDF-1α/CXCR4 axis in MM (Table 1).

### 4.1. Plerixafor

Plerixafor, a small-molecule bicyclam, reversibly binds to CXCR4 and antagonizes SDF-1α interaction. As previously described, this antagonist inhibits the migration and homing of MM cells in vitro and in vivo. The plerixafor could be used as a chemosensitizer in MM treatment because it disrupts their adhesion to the BM microenvironment and reverses resistance to antimyeloma agents. Ghobrial et al. reported the results from a phase I/II trial of plerixafor in combination with bortezomib as a chemosensitization strategy in relapsed/refractory MM (RRMM; NCT00903968) [101]. Phases I and II studies aim to assess the safety and maximum tolerated dose (MTD) and evaluate the treatment-related adverse events and response rate of the combination. Moreover, 58 patients were enrolled in this study. The median age was 63 years (range, 43–85 years). The MTD was plerixafor (0.32 mg/kg) and bortezomib (1.3 mg/m^2^). The overall response and clinical benefit rates were 48.5% and 60.6%, respectively. The median disease-free survival was 12.6 months. Moreover, the effect of plerixafor and bortezomib on the mobilization of plasma cells and HSCs in the peripheral blood was evaluated in this study. The CyTOF analysis showed significant mobilization of plasma cells, CD34^+^ stem cells, and immune T cells in response to plerixafor [101]. These results suggest that therapeutic targeting of the BM environment may overcome therapy resistance.

### 4.2. Ulocuplumab, BMS-936564/MDX-1338

Ulocuplumab is a fully human IgG4 monoclonal antibody that specifically recognizes human CXCR4 and effectively blocks SDF-1α binding to CXCR4 [102]. Ulocuplumab induces the apoptosis of myeloma cell lines and inhibits tumor growth of MM xenograft models [102]. The findings led to clinical trials in patients with RRMM. Ghobrial et al. reported the results from a phase Ib/II trial of ulocuplumab plus lenalidomide or bortezomib plus dexamethasone in RRMM (NCT01359657) [103]. Furthermore, 46 patients were enrolled (30 and 16 patients received ulocuplumab in combination with lenalidomide plus dexamethasone (Arm A) and combination with bortezomib plus dexamethasone (Arm B), respectively). The phase Ib/II study used a 3 + 3 design of that combination or the phase I dose escalation part and a two-stage outcome design to assess the efficacy and tolerability of ulocuplumab in combination with lenalidomide or bortezomib plus dexamethasone. The primary endpoint of phases I and II studies was to evaluate the safety and MTD of the combination therapies and determine the response rates of these combinations, respectively. Furthermore, 30 and 16 patients were enrolled in Arm A and Arm B, respectively. The median age was 60 years (range, 53–67 years). Moreover, the median number of prior therapy lines was three (range, 1–11). Ulocuplumab was administered at three dose levels (1, 3, and 10 mg/kg/dose). Consequently, no dose-limiting toxicity or MTD was identified. In most common treatment-related adverse events, neutropenia and thrombocytopenia were seen in 43.3% and 37.5% in Arm A and Arm B, respectively. No study on drug-related mortality was observed. The response (PR or better) and clinical benefit rates were 55.2% and 72.4% in Arm A, respectively. Both combination regimens were generally well-tolerated, with a high response rate, especially in combination with lenalidomide plus dexamethasone in patients with RRMM [103].

### 4.3. F50067

F50067 is a humanized monoclonal IgG1 anti-CXCR4 antibody and exerts antitumor effects via reducing the interaction of MM cells with the BM microenvironment and inducing antibody-dependent cellular cytotoxicity and compliment-dependent cytotoxicity. Fouquet et al. reported a phase I dose escalation study of F50067 alone and in combination with lenalidomide and low-dose dexamethasone (Len-dex) in RRMM [104]. Moreover, 14 patients with RRMM were enrolled in the study. Consequently, 10 or 4 patients received F50067 alone or in combination with Len-dex, respectively. Hence, MTD could not be established. Thrombocytopenia and neutropenia were observed in 100% and 92.9% of patients, respectively. The overall response and disease control rates were 66.7% and 33.3% in the combination and single agent groups, respectively. The study was discontinued due to hematological toxicities.

### 4.4. ^177^Lu- and ^90^Y-Pentixather

^68^Ga-pentixafor is a high-affinity CXCR4-targeted nuclear probe for positron emission tomography (PET) imaging [105,106]. ^68^Ga-pentixafor PET provided images with excellent specificity and contrast [106]. On the basis of such promising experiences, ^177^Lu- and ^90^Y-pentixather were developed [107,108]. Herrmann et al. reported the first in-human experience in three patients heavily pretreated with intramedullary and EMDs of MM who received CXCR4-directed endoradiotherapy [108]. Pretherapeutic ^177^Lu-pentixather dosimetry was performed before pentixather treatment. Patients then received additional chemotherapy and ASCT. A remarkable therapeutic effect was obtained in two patients. Complete response of all extramedullary lesions was observed in one patient. No acute adverse events occurred during or within one week after pentixather treatment. Hence, CXCR4-directed endoradiotherapy is feasible and has a promising RRMM response; further investigation of this therapy as a treatment option in heavily pretreated patients with MM, especially with EMD, is warranted.

### 4.5. Olaptesed Pegol, NOX-A12

Olaptesed pegol is a pegylated l-oligoribonucleotide that specifically binds and neutralizes SDF-1 [109]. Roccaro et al. showed that SDF-1 is highly expressed in active MM and in BM sites of tumor metastasis [110]. Furthermore, the authors demonstrated that SDF-1 neutralization within BM niches leads to a microenvironment that is less receptive for MM cells and reduces the homing and growth of clonal plasma cells and dissemination from bone-to-bone in in vivo murine and xenograft mouse models [110]. Interestingly, olaptesed pegol-dependent neutralization of SDF-1 inhibited MM tumor progression and prolonged survival compared with AMD3100-treated mice. The authors also demonstrated that olaptesed pegol chemosensitizes MM cells to bortezomib despite it having no single-agent activity on the tumor cells. These findings indicate that olaptesed pegol represents an agent that targets the interaction between BM niches and tumor cells, thereby disrupting BM colonization by MM cells. These results led to clinical trials evaluating the pharmacokinetics, pharmacodynamics, safety, and efficacy of olaptesed pegol in patients with RRMM [111]. Combining SDF-1 inhibition with bortezomib and dexamethasone (VD) was investigated in 28 patients with RRMM. Olaptesed pegol was given 1–2 h prior to bortezomib at doses of 1 mg/kg in cycle 1, 2 mg/kg in cycle 2, and 4 mg/kg in cycles 3–8. Bortezomib was given on days 1, 4, 8, and 11 of each 21-day cycle at a dose of 1.3 mg/m^2^. Oral dexamethasone (20 mg) was added on the day of and the day after bortezomib administration. The pharmacodynamic effects were observed 1 h after administration of olaptesed pegol. CD38+ CD138+ plasma cells and CD38+ CD138+ CD56+ CD19– myeloma cells were mobilized up to three-fold increases compared with baseline values in the peripheral blood [111]. The response (PR or better) was obtained in 19 of 28 patients (68%), which was better than those yielded in the clinical trials of CXCR4 inhibitors plerixafor [101] and ulocuplumab [103] in combination with VD. The median PFS and OS were 7.2 months and 28.3 months, respectively. On the other hand, treatment with olaptesed pegol was well tolerated and did not result in relevant additional toxicity when combined with VD [108]. Further clinical investigation of this novel inhibitor of SDF-1 is warranted.

**Table 1:** Overview of some compounds targeting SDF-1α/CXCR4 axis in MM cells.

Compound	Mechanism of Action	References
Plerixafor	CXCR4 antagonist Inhibits migration and homing of MM cells	[101]
Ulocuplumab	CXCR4 antagonist Induces apoptosis in MM cells with high CXCR4 expression Inhibits SDF-1α-induced migration	[102,103]
F50067	CXCR4 antagonist Inhibits cell migration and proliferation Antibody-dependent cellular cytotoxicity Compliment-dependent cytotoxicity	[104]
^177^Lu- and ^90^Y-pentixather	CXCR4-directed endoradiotherapeutic agent	[108]
Olaptesed pegol	SDF-1 inihibitor Neutralizes SDF-1 Inhibits colonization and dissemination of MM cells	[110,111]

## 5. Conclusions

Extensive basic, translational, and clinical research has uncovered the pivotal role of the SDF-1α/CXCR4 pathway in myeloma biology. Drug resistance and temporal and spatial tumor heterogeneity are closely associated with refractoriness of active myeloma. A therapeutic approach targeting this pathway should be further explored because SDF-1α/CXCR4 crosstalk between MM cells and BM microenvironment contributes to drug resistance, migration, and dissemination of MM cells. Future studies regarding the development of combined therapy with direct antimyeloma and inhibitory effects against tumor cell dissemination are necessary.

## Data Availability

Not applicable.
